# Effects of octreotide and insulin on colon cancer cellular proliferation and correlation with hTERT activity

**DOI:** 10.18632/oncoscience.58

**Published:** 2014-06-30

**Authors:** Georgios D. Ayiomamitis, George Notas, Apostolos Zaravinos, Ioannis Drygiannakis, Maria Georgiadou, Ourania Sfakianaki, Niki Mastrodimou, Kyriaki Thermos, Elias Kouroumalis

**Affiliations:** ^1^ Laboratory of Gastroenterology, School of Medicine, University of Crete, Heraklion, Greece; ^2^ 2nd Department of Surgery, Tzaneion General Hospital, Piraeus, Greece; ^3^ Laboratory of Experimental Endocrinology, School of Medicine, University of Crete, Heraklion, Greece; ^4^ Laboratory of Clinical Virology, School of Medicine, University of Crete, Heraklion, Greece; ^5^ Department of Laboratory Medicine, Karolinska Institute, Stockholm, Sweden; ^6^ Laboratory of Pharmacology, School of Medicine, University of Crete, Heraklion, Crete, Greece; ^7^ Department of Gastroenterology and Hepatology, School of Medicine, University of Crete, Heraklion, Greece

**Keywords:** Octreotide, insulin, colorectal cancer, hTERT activity, protein tyrosine phosphatases, sodium orthovanadate

## Abstract

Peptide hormone somatostatin and its receptors have a wide range of physiological functions and play a role in the treatment of numerous human diseases, including colorectal cancer. Octreotide, a synthetic somatostatin-analog peptide, inhibits growth of colonic cancer cells primarily by binding to G-protein coupled receptors and elicits cellular responses through second-messenger systems. Insulin also initiates mitogenic signals in certain cell types. The objective of the present study was to explore the effects of octreotide with or without insulin treatment, on Caco-2 and HT-29 human colon-cancer cell proliferation and to correlate their effects with the activation of telomerase reverse transcriptase (hTERT). The involvement of protein tyrosine phosphatases in the regulation of the anti-proliferative effect of octreotide was also evaluated. Sodium orthovanadate was used to reverse the anti- proliferative effect of octreotide. Telomerase activity was determined for each time point under octreotide and/or insulin treatment. Elevated expression of sst_1_, sst_2_ and sst_5_ was confirmed in both cell lines by RT-PCR. Immunocytochemistry detected sst_1_, sst_2A_, sst_2B_, sst_3_, sst_4_ and sst_5_ protein expression in the membranes of both cell lines. Octreotide inhibited the proliferation of Caco-2 and HT-29 cells in a time and dose-dependent manner. Insulin exerted proliferative effects in Caco-2 cells and octreotide reversed its effect in both cell lines. Sodium orthovanadate suppressed the anti-proliferative effect of octreotide both in Caco-2 and HT-29 cells. Telomerase activity was significantly reduced when Caco-2 cells were exposed to octreotide, under serum-free cultured medium. On the other hand, telomerase attenuation after octreotide treatment could not counteract the actions of insulin on both cells. Our data indicate that the use of octreotide could provide a possible therapeutic approach to the management of certain patients who suffer from colon cancer.

## INTRODUCTION

Colorectal cancer is one of the most common malignancies encountered in the western world and the third most common cause of cancer-related mortality. Its increasing incidence and associated morbidity and mortality reflect that colorectal cancer has been the subject of much research with regards to its etiology, diagnosis and treatment. Much has been learnt about the molecular biology of the disease in the last decades, thus paving the way for the possibility of the development of new therapeutic strategies.

Octreotide is an octapeptide that mimics natural somatostatin pharmacologically, though it is a more potent inhibitor of growth hormone, glucagon, and insulin than the natural hormone. Somatostatin was originally described as a natural growth-hormone-release inhibiting factor but it was later proved to have many metabolic and immunological effects through binding to five somatostatin receptors (ssts) [1, 2]. These receptors are G-protein coupled receptors and elicit cellular responses through second-messenger systems. These include both “direct” mechanisms and “indirect” mechanisms that might be the result of reduced or inhibited secretion of growth-promoting hormones and growth factors that stimulate the growth of various types of malignancies [3, 4]. Somatostatin and its synthetic analogue, octreotide, are potentially active against colorectal carcinoma due to their anti-proliferative and apoptosis-inducing activity. Insulin is a peptide hormone produced by the beta pancreatic cells, and it has also been shown to initiate mitogenic signals in certain cell types, acting as a trophic factor in tumor cells.

Telomerase is a nuclear ribonucleoprotein enzyme complex whose activity may be linked to the processes governing cellular senescence and cellular immortalization [5, 6]. Telomerase activity is typically absent in most normal human cells, but aberrantly expressed in human cancer cells [7]. Colorectal adenocarcinoma has been demonstrated to exhibit high levels of telomerase activity [8-10] and human telomerase reverse transcriptase (hTERT) has been proposed as a potential biomarker for colorectal cancer [9]. Recently, the inhibition of telomerase in actively dividing tumor cells was shown to lead to massive cell death [11]; however the regulatory processes governing the activation of telomerase expression and its level are still areas of active research.

The aim of this study was to evaluate the presence of somatostatin receptors on the colon cancer cell lines Caco-2 and HT-29, and to subsequently study the effects of the somatostatin analogue octreotide on cellular proliferation with or without the trophic effect of insulin. We also investigated the involvement of protein tyrosine phosphatases (PTPs) and telomerase activity in cellular proliferation.

## RESULTS

### Octreotide suppressed proliferation of colonic epithelial cells

**Figure 1:**
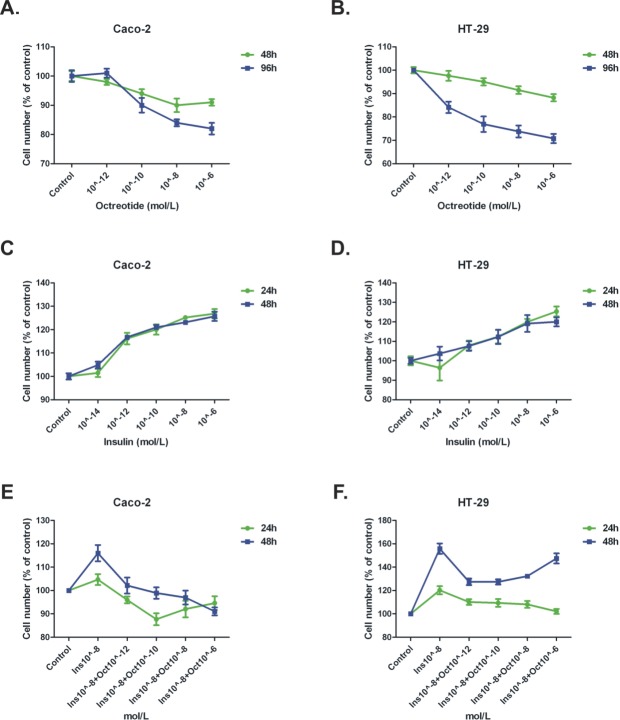
MTT cell proliferation assay on Caco-2 and HT-29 cells Time-course, octreotide dosing (A-B), insulin dosing (C-D), combination treatment (Insulin and octreotide dosing) (E-F).

Octreotide, a somatostatin analogue with a longer half life than somatostatin, inhibited the growth of Caco-2 in a dose-dependent way, starting from concentrations as low as 10^−10^M. This effect was more prominent at 96 h compared to 48 h of incubation (p<0.001) (Figure 1.A). HT-29 proliferation was also decreased both at 48 h and 96 h in a dose-dependent manner (p<0.001) (Figure 1.B).

### Insulin enhanced the proliferation of colonic epithelial cells

On the other hand, insulin significantly enhanced the proliferation of Caco-2 cells in a dose-dependent way (p<0.001) (Figure 1.C). Insulin's proliferative effect on HT-29 cells was noticeable at higher concentrations (10^−6^M) at 24 h of treatment (p<0.001) (Figure 1.D).

### Effect of insulin and octreotide co-incubation on colon cancer cells proliferation and PTP inhibition

**Figure 2:**
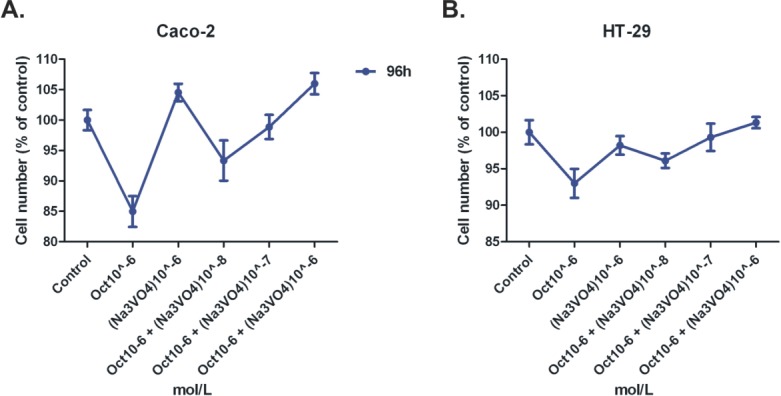
MTT cell proliferation assay on Caco-2 (A) and HT-29 (B) cells Time point 96h, octreotide and orthovanadate (Na_3_VO_4_) dosing.

Octreotide and insulin were then combined at various octreotide concentrations and various time points. We chose insulin concentration (10^−8^ M), for the combination experiments at all time points (24-72 h) because this concentration was previously shown to exhibit best proliferative effects on colonic epithelial cells. Octreotide was capable to reverse the proliferative effect of insulin at all possible concentrations tested, both at 24 h and 48 h of treatment (p<0.05) (Figure 1.E-F). When Na_3_VO_4_ was used in combination with octreotide in both cell lines, it could reverse the anti-proliferative effect of octreotide in a dose-depended manner (p<0.05), while alone it didn't show a significant effect on cellular proliferation (Figure 2.A-B).

### Expression of somatostatin and its receptors

**Figure 3:**
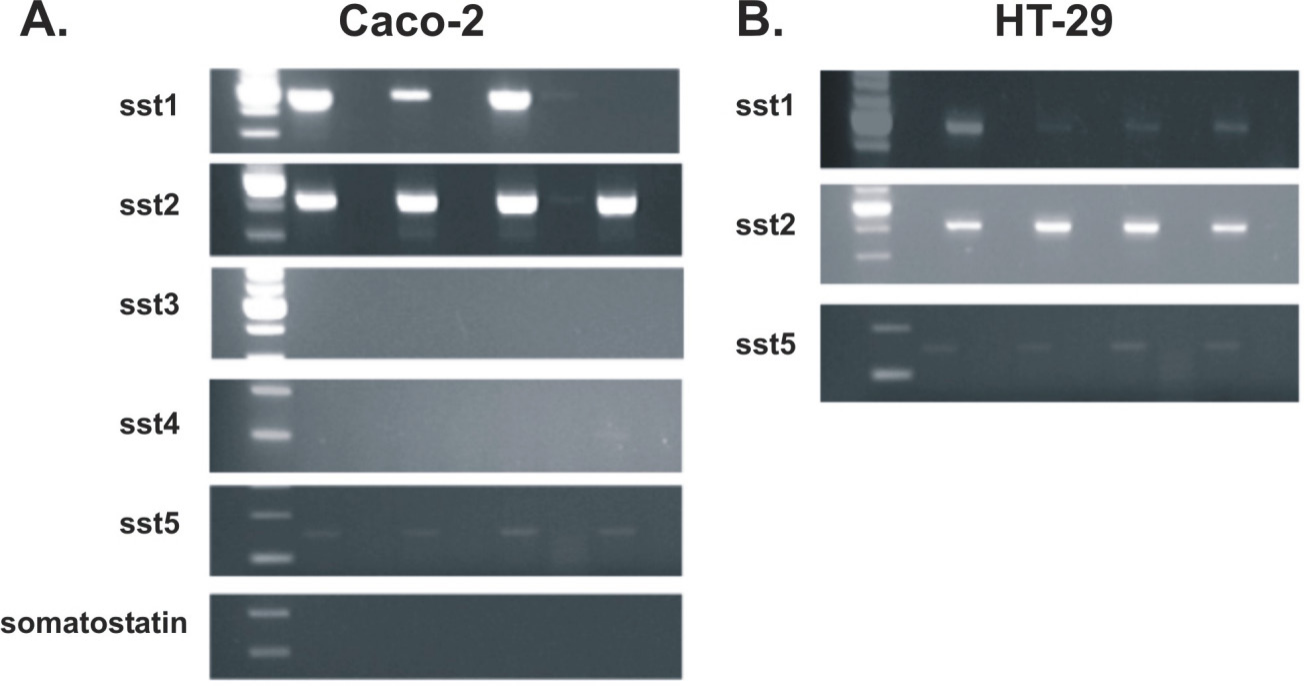
Sst mRNA expression on Caco-2 (A) and HT-29 (B) cells using RT-PCR Apart from sst3 and sst4 whose RNA levels were minimal, somatostatin, sst1, sst2 and sst5 were aberrantly expressed. Results from four different mRNA isolations.

**Figure 4:**
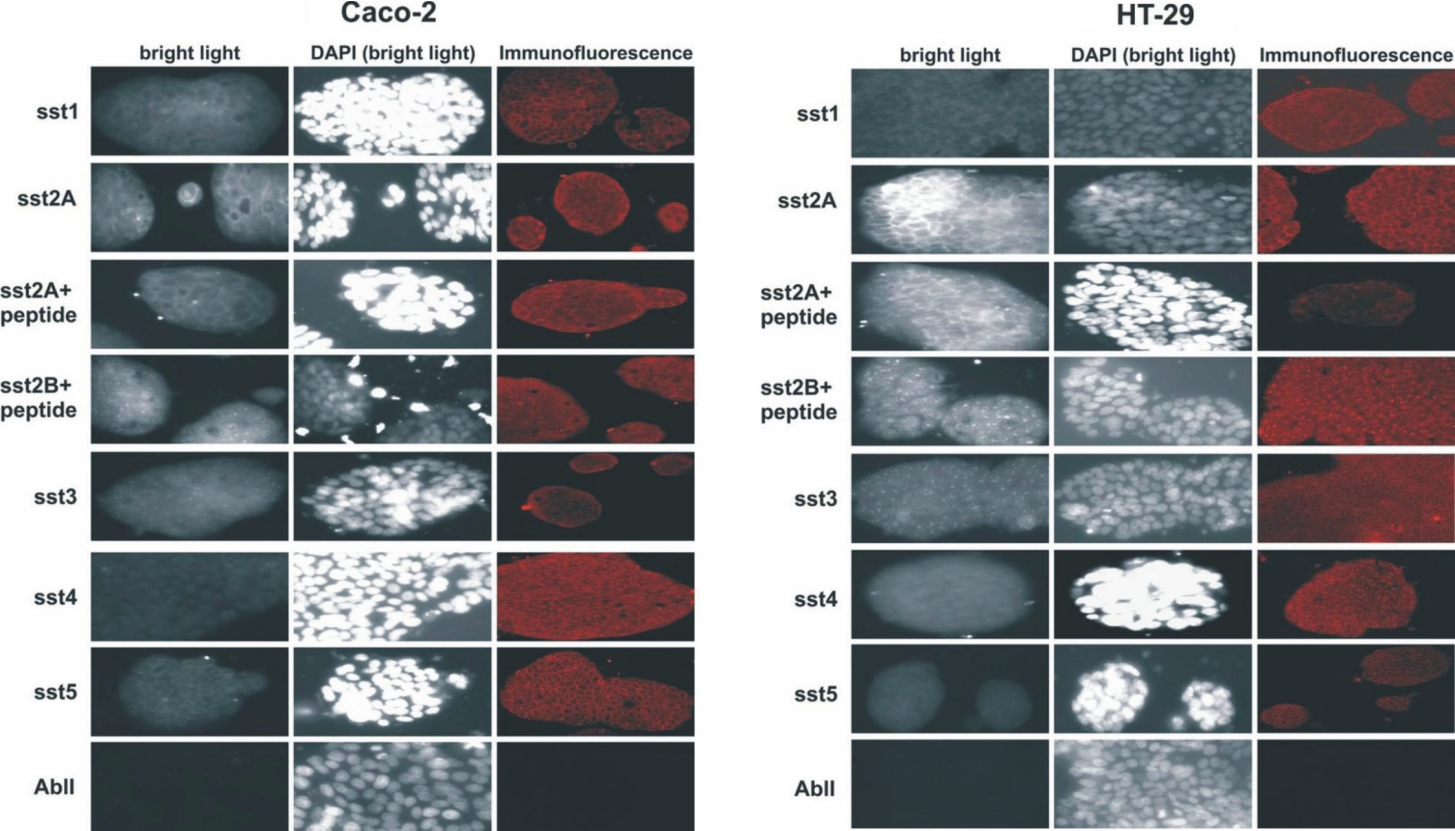
Immunocytochemistry for somatostatin receptors on Caco-2 and HT-29 cells Expression of sst1, sst2A, sst2B, sst3, sst4 and sst5 proteins was detected by Immunofluorescence in the membranes of both cell lines under confocal microscope.

The expression of somatostatin and its receptors, sst_1_, sst_2_, sst_3_, sst_4_ and sst_5_, was investigated at the RNA level in both cell lines. Apart from sst_3_ and sst_4_ whose RNA levels were minimal, somatostatin, sst_1_, sst_2_ and sst_5_ were aberrantly expressed (Figure 3.A-B). The expression of sst_1_, sst_2A_, sst_2B_, sst_3_, sst_4_ and sst_5_ proteins was detected by immunofluorescence in the membranes of both cell lines (Figure 4).

### Octreotide's effect on telomerase activity

In order to investigate the pathway that inhibits colonic epithelial-cell proliferation after treatment with octreotide, we studied the activity of telomerase in Caco-2 and HT-29 cells.

**Figure 5:**
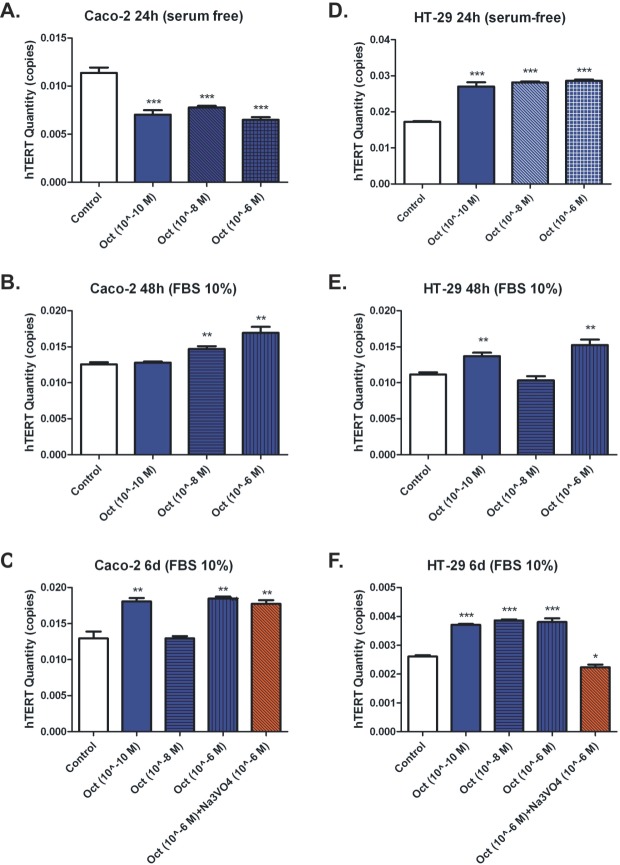
Telomerase activity measurement after octreotide treatment in serum-free (A,D) and 10% FBS supplemented culture medium in Caco-2 and HT-29 cells at 24h and 48h respectively (B, C, E and F) Telomerase activity measurement after octreotide dosing and orthovanadate (Na_3_VO_4_) treatment on 10% FBS treated Caco-2 and HT-29 cells at 6 days (C and F).

The telomerase activity was significantly reduced when serum-free Caco-2 cells were treated with octreotide (10^−10^-10^−6^ M, ~0.65-fold, p<0.001) (Figure 5.A). On the contrary, octreotide enhanced telomerase activity in Caco-2 cells cultured in the presence of 10% FBS after 48h (octreotide 10^−8^ M, 1.35-fold, p=0.004; octreotide 10^−6^ M, 1.47-fold, p=0.0027) (Figure 5.B). Enhancement of telomerase activity was noticed even 6 days post octreotide treatment (10^−10^ M and 10^−6^ M, 1.45-fold, p=0.0029 and p=0.0014). Surprisingly, sodium orthovanadate (10^−6^ M) in combination with 10^−6^ M octreotide did not seem to block octreotide's “enhancing” effect on telomerase (1.38-fold; p=0.0042) (Figure 5.C).

Interestingly, octreotide treatment (10^−10^-10^−6^ M) significantly enhanced telomerase activity in HT-29 cells cultured under serum free conditions (~1.5-fold, p<0.001) (Figure 5.D). Treatment with octreotide after 48h also enhanced telomerase activity in HT-29 cells cultured in the presence of 10% FBS (octreotide 10^−10^ M, 1.21-fold, p=0.003; octreotide 10^−6^ M, 1.33-fold, p=0.0025) (Figure 5.E). Treatment with octreotide after 6 days also enhanced telomerase activity (10^−10^-10^−6^ M, ~1.5-fold, p<0.001). Sodium orthovanadate (10^−6^ M) blocked the effect of octreotide (10^−6^ M) and dramatically reduced telomerase activity (0.86-fold, p=0.0105) (Figure 5.F).

## DISCUSSION

Somatostatin (somatotropin release-inhibiting factor - SRIF) and its analogue octreotide, act via six somatostatin receptors, (sst_1_, sst_2A_, sst_2B_, sst_3–5_) that belong to the super-family of transmembrane G-protein coupled receptors and are linked to several signal transduction pathways [2, 12]. While long-acting synthetic somatostatin analogues target the somatostatin receptors (sst), they differ in their binding affinity to these receptors [13], Somatostatin binds with high affinity to all somatostatin receptors, while octreotide is a preferential sst_2_ ligand, which targets the high levels of somatostatin receptor subtype 2 expressed in colon cancers [13]. Octreotide has also moderate affinity to sst_3_ and sst_5_ [14]. Recent studies have focused to sst signaling and its effects on cell growth. Sst_1,2,4_ and sst_5_ have been shown to cause cell cycle arrest, while sst_3_ and less so sst_2_ can induce apoptosis [15]. Furthermore sst_1,4_ and sst_5_ modulate the MAP kinase pathway and induce G1 cell cycle arrest, while sst_3_ and sst_2_ promote apoptosis by p53-dependent and independent mechanisms, respectively [16]. It appears that the presence of sst may be important for tumor response to octreotide treatment [17]. Due to its varied actions, somatostatin and its analogs can potentially contribute to cancer diagnosis and treatment through multiple mechanisms.

Compelling evidence has implicated somatostatin in the inhibition of the growth and development of various normal and tumor cells. Thus, somatostatin analogues show anti-neoplastic activity in a variety of experimental models in vivo and in vitro [18, 19]. Over the past decade, impressive anti-neoplastic effects of somatostatin and its analogs have been reported in several tumor models and cancer cell types [20-26]. A similar anti-proliferative effect of SRIF and its analogues has also been reported in numerous in vitro systems [16, 27-29]. SRIF and octreotide are successfully used for the treatment of neuroendocrine tumours and exert anti-proliferative effects on several cell types [30-39].

In the present study, we observed inhibition of growth in two colon cancer cell lines (Caco-2 and HT-29), after treatment with octreotide in a time and dose-dependent manner. To our knowledge, this is the first reported description of such an effect. Our results showed that inhibition of growth in Caco-2 cells when treated with octreotide, depressed telomerase activity; whereas in 10% FBS culture medium, octreotide exerts opposite effects by enhancing the activity of telomerase. We also showed that HT-29 cells irrespective the cultured medium, exhibit unexpected enhancement of telomerase activity after treatment with octreotide.

Thus, we speculate that octreotide might inhibit cellular proliferation selectively in the Caco-2 cells by reducing the telomerase activity, whereas on the HT-29 cells, it seems to inhibit cellular proliferation through different molecular pathways. As previously suggested by Gao S. *et al.* [40], one of them might be the AKT/ PI3K pathway. Furthermore, Wang et al. [41] showed that octreotide inhibits the growth of colonic cancer SW480 cells through modulation of the Wnt/β-catenin pathway. Their findings revealed a negative regulation of the Wnt/β- catenin pathway by peptide hormone G protein-coupled receptors. The results of Chen et al. [42] also showed that octreotide can inhibit human colonic cancer cell growth through inhibition of Wnt/beta-catenin signaling pathway. On the other hand when Caco-2 cells were exposed to octreotide in the presence of 10% FBS, the activity of telomerase was enhanced. The same effect could also be observed in HT-29 cells. Thus, when Caco-2 cells are cultured in heat-inactivated 10% FBS medium, it seems that they exert different properties than when cultured in serum-free medium. Interestingly the same could not be observed in HT-29 cells where octreotide treatment enhances telomerase activity, independent of the cultured medium.

Activation of protein tyrosine phosphatases (PTPs) is one of the mechanisms via which somatostatin mediates its anti-proliferative effects [16, 27]. In order to investigate whether PTPs are involved in the intracellular pathway regulating the anti-proliferative effect of octreotide on HT-29 and Caco-2 cells, we studied whether the blockade of PTPs could reverse the anti-proliferative effect of octreotide. As expected, the PTP inhibitor sodium orthovanadate, caused a dose-depended attenuation of the effect of octreotide on both cell lines. Yet, sodium orthovanadate alone did not have any effect on the cellular proliferation of either colon cancer cell lines. It is clearly apparent that the anti-proliferative effect of octreotide on colon cancer cells is mediated by PTPs. Similar results were reported in pancreatic cancer cell lines with the somatostatin analogue TT-232 [13, 29].

However, this might not be the only underlying mechanism. Octreotide inhibition of gastric cancer cells has been reported to be associated with telomerase activity [40]. Therefore we assessed telomerase activity of our cancer cell lines after incubation with octreotide. The results were different in the two cell lines. Octreotide profoundly inhibited telomerase activity in CaCo2 cells but increased activity in the HT-29 cells. In colon cancer, telomerase has been reported to be activated very early in the process of this disease [43], which suggested that activation of telomerase might be also a determining factor contributing to the tumorigenesis. It is not clear from the present study why this discrepancy occurred. Nonetheless, it is a probable explanation why HT-29 are more resistant to the action of octreotide.

Telomerase is an RNA-dependent DNA polymerase comprised of an RNA component [44] that serves as a template; the catalytic subunit, human telomerase reverse transcriptase (hTERT); and a telomerase-associated protein, of unknown function [5]. Telomerase uses its RNA template to catalyze the addition of TTAGGG repeats to the ends of vertebrate chromosomes [45]. In the absence of telomerase, the telomere will shorten with each successive cell division. This occurs because DNA polymerase α is unable to replicate the very ends of linear DNA, thus leading to the progressive shortening of the telomeric ends in normal somatic cells and appears to be linked to the limited proliferative capacity of normal cells [46]. Telomerase activity has been detected in over 90% of human cancers [44, 47, 48].

In colon cancer, telomerase is activated very early in the process of the disease [49], suggesting that its activation might be also a determining factor that contributes to the process of tumorgenesis. In the present study, we found that the activity of telomerase, measured in hTERT copies, was reduced after octreotide treatment under serum-free conditions in Caco-2 colon cancer cells. Surprisingly, we observed opposite effects on the activity of telomerase after octreotide treatment under serum-free conditions in HT-29 cells.

Hu et al. (2004) [43] studied the expression of sst3 protein in various gastric cancer cell lines. Using immunofluorescence and western blot analysis revealed that sst3 protein was expressed more in GES and SGC7901 cells than in AGS cells and no expression was found in MKN45 cell. In our study, Caco-2 and HT-29 cells behaved differently and exhibited different properties on telomerase activity after octreotide treatment. This can be well explained due to possible different sst subtypes expression profiles between these two cell-lines, thus resulting in different telomerase activity in serum-free cultured conditions.

On the contrary, after octreotide treatment, telomerase activity was surprisingly enhanced on both Caco-2 and HT-29 cell-lines in 10%FBS cultured medium. The culture medium (in particular, insulin) might play an important role affecting the effect of octreotide. Another explanation might be that octreotide is less active on colon cancer cells. Pawlikowski M *et al*. (1998) [50], studied the differential effects of somatostatin and octreotide on pituary tyrosin kinase (PTK), and showed that in the case of colonic cancer, the native somatostatin was more effective in inhibiting PTK than octreotide which is active only at the highest concentration studied (10-5 M) and surprisingly is much less active in comparison to native somatostatin. It seems that colonic tumors express less abundantly the octreotide-sensitive subtypes of somatostatine receptors. It is worth to recall that in contrast to octreotide, the native somatostatin binds approximately with the same affinity to all subtypes of sst-receptors [50].

When the PTP inhibitor sodium orthovanadate was applied, it caused attenuation of telomerase activity by completely reversing the action of octreotide in HT- 29 cells cultured in the presence of 10% FBS. However, to our surprise this was not also observed in the Caco-2 cells. This indicates that under certain circumstances, HT- 29 and Caco-2 cells behave differently as far as telomerase activity is concerned. This observation implies that these two cell lines do not derive from the same organ. Although both of them were isolated from primary colonic tumors, they derive from different patients (Caco-2 cells derive from a female patient of 44 years old; whereas HT-29 cells derive from a male patient of 72 years old) and most probably were isolated from different locations in the colon. There is evidence today that considers colorectum as two or three different organs, due to the different biological profile and properties between different colon locations, rather than a single unique organ. This might explain the interesting phenomenon of different properties between Caco-2 and HT-29 colon cancer cell lines.

Insulin and the insulin-like growth factors (IGF-1 and IGF-2) represent a family of hormones/growth factors that regulate metabolism, growth, cell differentiation and survival of most tissues in mammals. Insulin and IGF-1 initiate their action via highly homologous signaling systems. The insulin and IGF-1 receptors are members of the tyrosine kinase family of receptors [51]. The mechanisms governing telomerase activation are incompletely understood. Several studies have suggested a link between serum IGF-I levels and risk of several cancers, including prostate, breast, colorectal, and lung carcinoma [52-55]. Lawrence *et al.* (2003) [56], found that IGF-I could potentially contribute to the immortalization process of malignancy by up-regulating telomerase activation, leading to telomere lengthening and extension of the cellular life span of prostate cancer cells [56]. Sophie Baron-Delage *et al.* (1994) [57], showed that insulin has a potent mitogenic effect on Caco-2 cells which is specifically mediated by its own receptors. There is evidence that insulin is able to activate a downstream effector molecule of the mitogenic pathway in this cell type by inducing a rapid and sustained stimulation of MAP kinase activity [57].

Baring all these in mind we can well explain the results of our study, where Caco-2 and HT-29 cell-lines seem to exert different properties on telomerase activation after octreotide treatment, when cultured in heat-inactivated 10% FBS medium, than when cultured in serum-free medium. The heat-inactivated 10% FBS cultured medium includes insulin, which has a potent mitogenic/proliferation effect on colon cells. The attenuation of telomerase activity that is observed in serum-free conditions after octreotide treatment, was totally reversed when the cultured medium was changed to heat-inactivated 10% FBS. Surprisingly, then the telomerase activity was enhanced despite the anti-proliferative action of octreotide. It seems that the depression caused by octreotide cannot overcome the mitogenic effect of the cultured medium (i.e. insulin/IGF) on the activity of telomerase in both cell lines.

## CONCLUSIONS

We suggest that octreotide acts on cell proliferation mainly via PTP or telomerase signaling and its use could provide an effective therapeutic approach to the management of certain patients suffering from colon cancer. These observations need to be further confirmed.

## MATERIALS AND METHODS

### Cell lines, reagents and chemicals

The human colon cancer epithelial cell-lines Caco-2 and HT-29 were obtained from the European Collection of Animal Cell Cultures (ECACC, Porton Down, UK). Octreotide and insulin were provided by Sandoz Corp. (Wilson, NC) The reagents McCoy's 5A, fetal bovine serum (FBS), penicillin, streptomycin, L-glutamine, Modified Eagles Minimum Essential Medium (MEM) and MEM non-essential amino acids, sodium bicarbonate and sodium pyruvate were all purchased from Gibco (Invitrogen, UK). The chemicals 3-(4,5-dimethyl thiazol- 2-yl)-2, 5-diphenyl tetrazolium bromide (MTT), sodium orthovanadate and dimethyl sulfoxide (DMSO) were provided by Sigma-Aldrich (Sigma-Aldrich Chemie GmbH, Germany). A Bio-tek microplate reader (Bio-tek Instruments, Inc, USA) was used for the MTT assays. All plastic-wares were purchased from NUNC (NUNC, Roskilde, Denmark).

### Cell culture

Caco-2 and HT-29 cells were cultured at 75 cm^2^ flasks with MEM and McCoy's 5A medium, respectively. Both media were enriched with 10% heat-inactivated FBS, 100 U/ml penicillin, 100 μg/ml streptomycin, 2 mM L-glutamine, 0.1 mM MEM non-essential amino acids, 1.5 g/L sodium bicarbonate and 1.0 mM sodium pyruvate. Cells were maintained in humidified atmosphere at 37°C and 5% CO_2_. Culture medium was changes every 3 days.

### 3-(4,5-imethyl thiazol-2-yl)-2, 5-diphenyl tetrazolium bromide (MTT) assay

MTT assays were used to determine cell viability and proliferation, as previously described [58]. In brief, Caco-2 and HT-29 cells were seeded at 2×10^4^ cells/well and 1.5×10^4^ cells/well, respectively, in 24-well plates and incubated in culture medium overnight (day 0). Then Caco-2 and HT-29 cells were made quiescent by serum deprivation and treated with 10^−6^, 10^−8^, 10^−10^ and 10^−12^ mol/L of octreotide and/or 10^−6^, 10^−8^, 10^−10^, 10^−12^ and 10^−14^ mol/L insulin, respectively. Sodium orthovanadate (Na_3_VO_4_) was also used in various concentrations (10^−6^-10^−8^ M) in combination with octreotide. Sodium orthovanadate preserves protein phosphorylation by inhibiting endogenous phosphatases that are present in the cell lysate mixture. Sodium orthovanadate was added to prove that the anti-proliferative effect of octreotide on the cells is mediated via the inhibition of endogenous phosphotyrosine phosphatases (PTPs). Caco-2 and HT-29 cells were treated with equal amounts of normal saline, which served as control. After 72 h of incubation, 500 μl of MTT (2.5 mg/ml) was added into each well. After 4 h of incubation, 150 μl DMSO was added to each well. The plate was mixed gently by rocking back and forth until the blue sedimentation crystals were completely dissolved. Finally, 200 μl of each sample was transferred to a 96-well plate and the absorbance was read on a microplate reader at 540 nm with a reference wavelength at 630 nm. Each treatment was performed in triplicate.

### Telomeric Repeat Amplification Protocol (TRAP)

The TRAP in vitro assay was used in order to detect telomerase activity in both cell lines, according to the manufacturer's instructions (Quantitative Telomerase Detection Kit-US Biomax, Inc.). Viable cells were lysed and the telomerase activity in the cell extract was determined through its ability to synthesize telomeric repeats onto an oligonucleotide substrate in vitro upon the addition of the appropriate buffer conditions and dNTPs. Telomerase from the cell extract adds telomeric repeats onto a substrate oligonucleotide and the resultant extended products were subsequently amplified by the qPCR and visualized using SYBR Green dye. All qPCR experiments were conducted on the Mx3000P real-time PCR thermal cycler using the software version 2.00 (Stratagene, La Jolla, CA). For the estimation of telomerase activity, a positive control (TSR template) was used in order to generate a standard curve, which was consisted of eight serial dilutions that ranged from 0.5 μg/μl (3×10^5^ molecules/reaction) to 6.4×10^−6^ μg/μl (4 molecules/ reaction).

### Reverse-transcription polymerase chain reaction (RT-PCR)

Total RNA was extracted from Caco-2 and HT- 29 cells using the TRIzol^®^ reagent. Genomic DNA was eliminated by RNA incubation in gDNA Wipeout Buffer at 42°C for 2 minutes and the RNA was then used directly in reverse transcription (RT). RT was performed with 1 μg of total RNA, Quantiscript Reverse Transcriptase, Quantiscript RT Buffer and RT Primer Mix, according to the supplier's instructions. (Qiagen, Crawley, UK). The primers for the somatostatin receptors were selected from the NCBI UNISTS databank and those for somatostatin, were used as previously described [59-61] (Table 1). All primers were synthesized and supplied by MWG (Ebersberg, Germany). Each set of primers was tested with at least three different RNA samples that were treated independently. The cDNA was used as template for PCR reaction using platinum Taq DNA polymerase (Invitrogen, UK). Amplification was performed using a Thermocycler (Biometra, Germany) as follows: 94°C for 2 min; 40 cycles of 94°C for 30s; 55-60°C for 60s; and 72°C for 3 min, followed by a final elongation period of 10 min at 72°C. GAPDH was also run as normalizer gene. No-template control PCR was also performed simultaneously with every reaction. The PCR products were separated and visualized in ethidium bromide-stained 2% agarose gel by electrophoresis.

**Table 1:** Primers of the somatostatin receptors sst_1-5_ and somatostatin that were used for the RT-PCR experiments

Gene name	Primer sequences
β-Actin	5′-GGTGGCTTTTAGGATGGCAAG-3′ 5′-ACTGGAACGGTGAAGGTGACAG-3′
Sst1	5′-CCACCAACATCTACATCCTA-3′ 5′-CCACCATCATCACCATTAAG-3′
Sst2	5′-CATCTTCTGCCTGACAGTC-3′ 5′-CCACCACAAAGTCAAACAT-3′
Sst3	5′-AGAACGCCCTCTCCTACTGG-3′ 5′-GTTGACGATGTTGAGCACG-3′
Sst4	5′-AACCTCGTCGTGACCAG-3′ 5′-AGCAGTGGCATAGTAGTCCAG-3′
Sst5	5′-GCTTCCAGAAGGTTCTGTGC-3′ 5′-TTGCTGGTCTGCATAAGCC-3′
Somatostatin	5′-GTTTCTGCAGAAGTCCCTGG-3′ 5′-AATTCTTGCAGCCAGCTTTG-3′

### Immunocytochemistry (ICC)

Caco-2 and HT-29 cells were grown on poly-L-lysine-coated coverslips overnight. After the appropriate treatment with octreotide, cells were fixed with 4% paraformaldehyde and 0.2% picric acid in phosphate buffer (pH 6.9) for 40 min at room temperature and washed several times. The specimens were permeabilized and then incubated with 1 μg/ml anti-sst1, anti-sst2A, anti-sst2b, anti-sst3, anti-sst4 or anti-sst5 antibodies, followed by cyanine-conjugated secondary antibodies (Amersham, Braunschweig, Germany). The cells were counter stained with 1 μg/ml DAPI for 1 min, rinsed with PBS, mounted and examined using a Leica TCS-NT laser scanning confocal microscope (Leica Microsystems, Nussloch, Germany) [62, 63].

### Statistical analysis

Results are shown as mean±standard error of the mean (SEM). All the experiments were performed in triplicates. The statistical significance of the difference between two groups was evaluated by two-tailed Student's t-test or Two-Way ANOVA followed by Bonferroni test correction, using GraphPad Prism 5 (Graph Pad, Software, La Jolla CA, USA). A p-value <0.05 was considered as threshold of significance.
